# The metabolic enzyme AdhE controls the virulence of *Escherichia coli* O157:H7

**DOI:** 10.1111/mmi.12651

**Published:** 2014-06-09

**Authors:** Katherine S H Beckham, James P R Connolly, Jennifer M Ritchie, Dai Wang, Jayde A Gawthorne, Amin Tahoun, David L Gally, Karl Burgess, Richard J Burchmore, Brian O Smith, Scott A Beatson, Olwyn Byron, Alan J Wolfe, Gillian R Douce, Andrew J Roe

**Affiliations:** 1 Institute of Infection, Immunity and Inflammation, College of Medical, Veterinary and Life Sciences, University of GlasgowGlasgow, G12 8TA, UK; 5 Institute of Molecular and Cell Biology, College of Medical, Veterinary and Life Sciences, University of GlasgowGlasgow, G12 8TA, UK; 7 School of Life Sciences, College of Medical, Veterinary and Life Sciences, University of GlasgowGlasgow, G12 8TA, UK; 2 Faculty of Health and Medical Sciences, University of SurreyGuildford, GU2 7XH, UK; 3 Immunity and Infection Division, The Roslin Institute and R(D)SVS, The University of EdinburghEaster Bush, Midlothian, EH25 9RG, UK; 4 Faculty of Veterinary Medicine, Kafrelsheikh University33516, Kafr el-Sheikh, Egypt; 6 School of Chemistry and Molecular Biosciences and Australian Infectious Diseases Research Centre, University of QueenslandSt. Lucia, Qld, 4072, Australia; 8 Department of Microbiology and Immunology, Loyola University Chicago, Stritch School of Medicine2160 S. First Ave., Bldg. 105, Maywood, IL, 60153, USA

## Abstract

Classical studies have focused on the role that individual regulators play in controlling virulence gene expression. An emerging theme, however, is that bacterial metabolism also plays a key role in this process. Our previous work identified a series of proteins that were implicated in the regulation of virulence. One of these proteins was AdhE, a bi-functional acetaldehyde-CoA dehydrogenase and alcohol dehydrogenase. Deletion of its gene (*adhE*) resulted in elevated levels of extracellular acetate and a stark pleiotropic phenotype: strong suppression of the Type Three Secretion System (T3SS) and overexpression of non-functional flagella. Correspondingly, the *adhE* mutant bound poorly to host cells and was unable to swim. Furthermore, the mutant was significantly less virulent than its parent when tested *in vivo*, which supports the hypothesis that attachment and motility are central to the colonization process. The molecular basis by which AdhE affects virulence gene regulation was found to be multifactorial, involving acetate-stimulated transcription of flagella expression and post-transcriptional regulation of the T3SS through Hfq. Our study reveals fascinating insights into the links between bacterial physiology, the expression of virulence genes, and the underlying molecular mechanism mechanisms by which these processes are regulated.

## Introduction

Enterohaemorrhagic *Escherichia coli* (EHEC) strains cause diarrhoea, haemorrhagic colitis and haemolytic uraemic syndrome (HUS), with the young and the elderly being most at risk (Nataro and Kaper, 1998). Cattle can be asymptomatically colonized by EHEC and thus can act as the major reservoir for human infections. Production of Shiga toxin (Stx) is responsible for HUS, the leading cause of acute paediatric renal failure in both the UK and USA (Noris and Remuzzi, 2005). No vaccines are currently available against EHEC infections and antibiotic treatment is associated with increased clinical severity, which can be attributed to increased production and/or release of the Stx toxin.

EHEC strains colonize the intestinal mucosa via a carefully regulated process that involves first expression of flagella and then expression of a Type Three Secretion System (T3SS) (Mahajan *et al.*, 2009). The flagellum is a key motility organelle, while the T3SS is an organelle used widely by Gram-negative bacteria to facilitate interactions with host cells, including invasion, host cell killing and, in the case of EHEC, close attachment (Coburn *et al.*, 2007). T3SS-mediated attachment of the bacterium to host epithelium is characterized by formation of distinctive attaching and effacing (A/E) lesions (Frankel *et al.*, 1998). In EHEC strain O157:H7, the T3SS is encoded by the locus of enterocyte effacement (*LEE*) (Elliott *et al.*, 2000). The first three operons (*LEE1–3*) encode a multi-protein apparatus that spans the bacterial inner and outer membranes (Hueck, 1998). The fourth operon (*LEE4*) encodes proteins for a needle complex (EscF), a translocation filament (EspA) and pore forming proteins (EspB/D) (Hartland *et al.*, 2000). *LEE5* contains the genes for the adhesins Tir and intimin (Sánchez-Sanmarti *et al.*, 2001).

In addition to motility, flagella mediate initial adhesion to epithelial cells *in vitro* and *in vivo* during colonization of the bovine intestine (Mahajan *et al.*, 2009) and, therefore, play a role in the initial stages of the infection process. However, the expression of this virulence factor is tightly regulated. Since flagellin is the main agonist of Toll-like receptor 5 (TLR-5) and thus induces the production of pro-inflammatory cytokines (Hayashi *et al.*, 2001), the ability to switch off expression of this virulence factor is important to evade detection by the immune system. Production of flagella requires expression of three classes of promoters (Chevance and Hughes, 2008). The class I operon includes genes for the master regulator FlhD_4_FlhC_2_, which binds to and activates class II promoters. One class II operon includes *fliA*, which encodes the flagella-specific sigma factor (σ^28^), which is required for the expression of class III gene expression. The class III operon encodes structural proteins (e.g. flagellin) and the chemotactic proteins. Regulation of the flagellar regulon is complex and sophisticated, controlled by many stimuli and regulators (Wolfe and Visick, 2008). Importantly, flagella and the T3SS are cross-regulated to prevent coexpression (Iyoda *et al.*, 2006), an arrangement that fits with their different functions – motility and initial attachment for the flagella (Mahajan *et al.*, 2009), followed by close attachment and cell subversion for the T3SS.

Our previous work identified a set of proteins that are implicated in the regulation of virulence in EHEC; one was AdhE (D. Wang *et al.*, 2011), a bi-functional acetaldehyde-CoA dehydrogenase and alcohol dehydrogenase. Most studies of AdhE have focused mainly on its role in ethanol production and its importance during anaerobic respiration (Clark and Cronan, 1980). In contrast, the role of AdhE under aerobic growth conditions has been poorly studied. However, two studies link AdhE to virulence in *Salmonella typhimurium*; a transposon insertion into *adhE* resulted in diminished capacity of *S. typhimurium* to survive murine macrophages (Baumler *et al.*, 1994), while deletion of *adhE* affected SPI-1-mediated gene expression and infectivity (Abernathy *et al.*, 2013). In this study, we found that deletion of the *adhE* from EHEC resulted in a stark pleiotropic phenotype: excretion of acetate into the surrounding environment, strong expression of non-functional flagella, suppression of the T3SS, and reduced binding to host cells. Further analysis of the Δ*adhE* mutant revealed insights into the molecular mechanism by which deletion of *adhE* affects gene expression. The identification of AdhE as a protein critical for proper regulation of virulence gene expression paves the way for further studies to specifically target this protein.

## Results

### AdhE affects virulence factor expression

**Fig 1:**
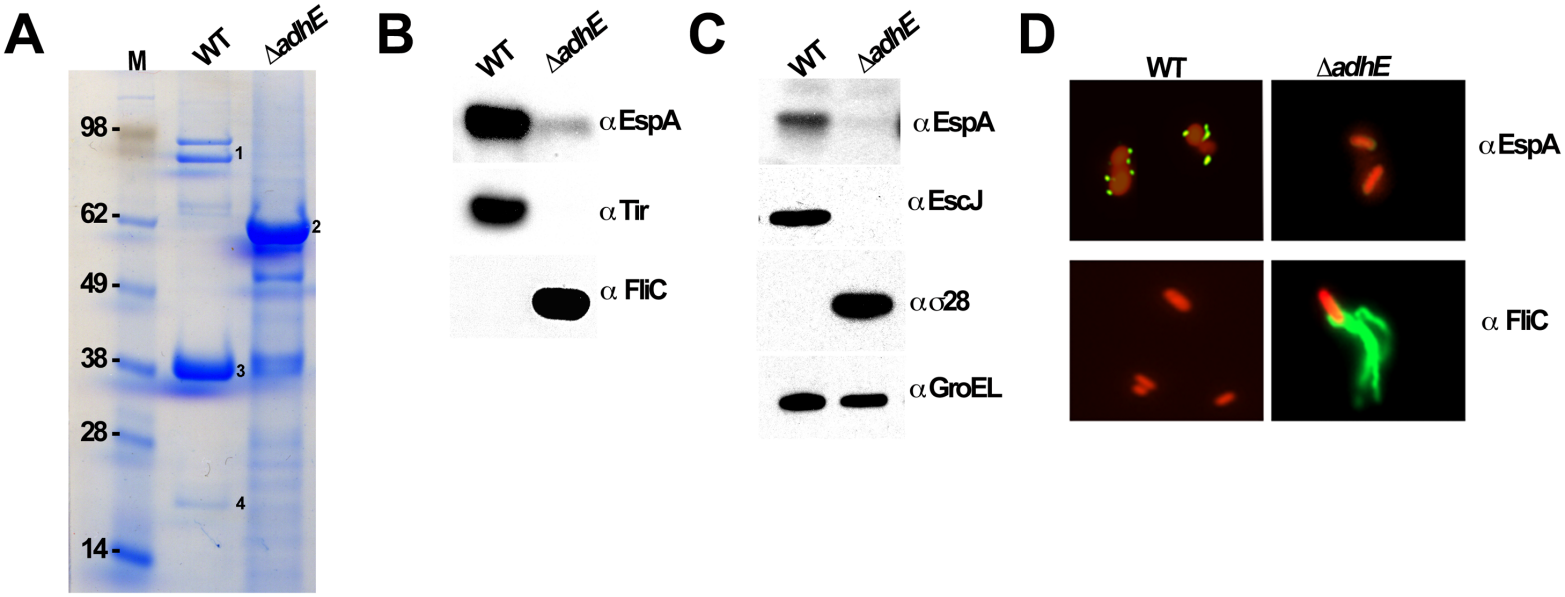
Deletion of *adhE* affects expression of the T3SS and flagella in *E. coli* O157:H7.A. The secreted protein profile of WT EHEC and Δ*adhE* bacteria following culture in MEM-HEPES media. Protein identity was confirmed by tandem MS analysis and the bands corresponding to Tir (1), FliC (2), EspD (3) and EspA (4) are indicated.B and C. (B) Immunoblot analysis of secreted proteins (EspA, Tir and FliC) and (C) cell lysates (EscJ, σ^28^ and GroEL) from WT EHEC and Δ*adhE* strains.D. Immuofluorescence microscopy of WT and Δ*adhE* bacteria transformed an RFP expressing plasmid (pRFP, red) probed with αEspA and αH7 (FliC) antibodies and Alexaflour 488 conjugated secondary antibodies (green). Scale bars are 2 μM.

To evaluate whether AdhE regulates virulence factor expression, an *adhE* deletion mutant in the EHEC strain O157:H7 was generated by allelic exchange. No difference in the growth rate was observed between the wild-type (WT) parent and its isogenic Δ*adhE* mutant. To assess any changes in the expression of secreted virulence proteins, bacteria were cultured in MEM-HEPES media (Roe *et al.*, 2003), and the secreted protein fraction was isolated. Secreted proteins were analysed by SDS-PAGE, tandem mass spectrometry (MS) and immunoblotting. The WT and Δ*adhE* mutant showed marked differences in their secreted protein profiles (Fig. 1A). Most starkly, the Δ*adhE* mutant increased expression of a ∼ 60kDa protein. Tandem mass-spectrometry identified this protein as FliC, the major structural subunit of the flagellar filament (Fig. 1A), a result confirmed by immunoblot analysis (Fig. 1B). Correspondingly, the Δ*adhE* mutant exhibited markedly elevated expression of σ^28^ (Fig. 1C), the flagellar-specific sigma factor required for *fliC* transcription. In contrast, the chaperone GroEL was expressed at similar levels in the WT and Δ*adhE* mutant (Fig. 1C). That these defects were the consequence of the *adhE* deletion was verified by allelic exchange with the WT allele; the resultant strain suppressed FliC secretion (Fig. S1) and fully restored secretion of effector proteins.

Given that flagella and T3SS expression are cross regulated, we sought changes in secreted effector proteins and found that T3SS-associated proteins, including EspA and Tir, were markedly reduced in the Δ*adhE* mutant (Fig. 1A and B). To determine if this phenotype was due to a defect in assembly of the T3SS, whole cell lysates from WT and Δ*adhE* were analysed for the presence of EscJ, one of the basal apparatus proteins of the T3SS. EscJ (Fig. 1C) was detected only in WT bacteria, suggesting expression of the entire T3SS was reduced. Since the effector and the T3SS apparatus proteins were not detected in the Δ*adhE* mutant (Fig. 1C), we conclude that the entire T3SS regulon is downregulated in the *adhE* mutant.

To visualize the pleiotropic phenotype, indirect immunofluorescence microscopy of EspA and FliC was performed. The Δ*adhE* mutant produced diminished levels of EspA protein, but increased levels of FliC (Fig. 1D). Indeed, the mutant had assembled multiple intact flagellar filaments on its surface. In summary, these data showed that the deletion of *adhE* results in a stark switch in phenotype: suppression of expression of the T3SS and strong upregulation of flagella. This was a surprising result, as the bacteria had been cultured under conditions that normally promote T3S and repress flagella production.

### The *ΔadhE* mutant displays a ‘paralysed’ phenotype

**Fig 2:**
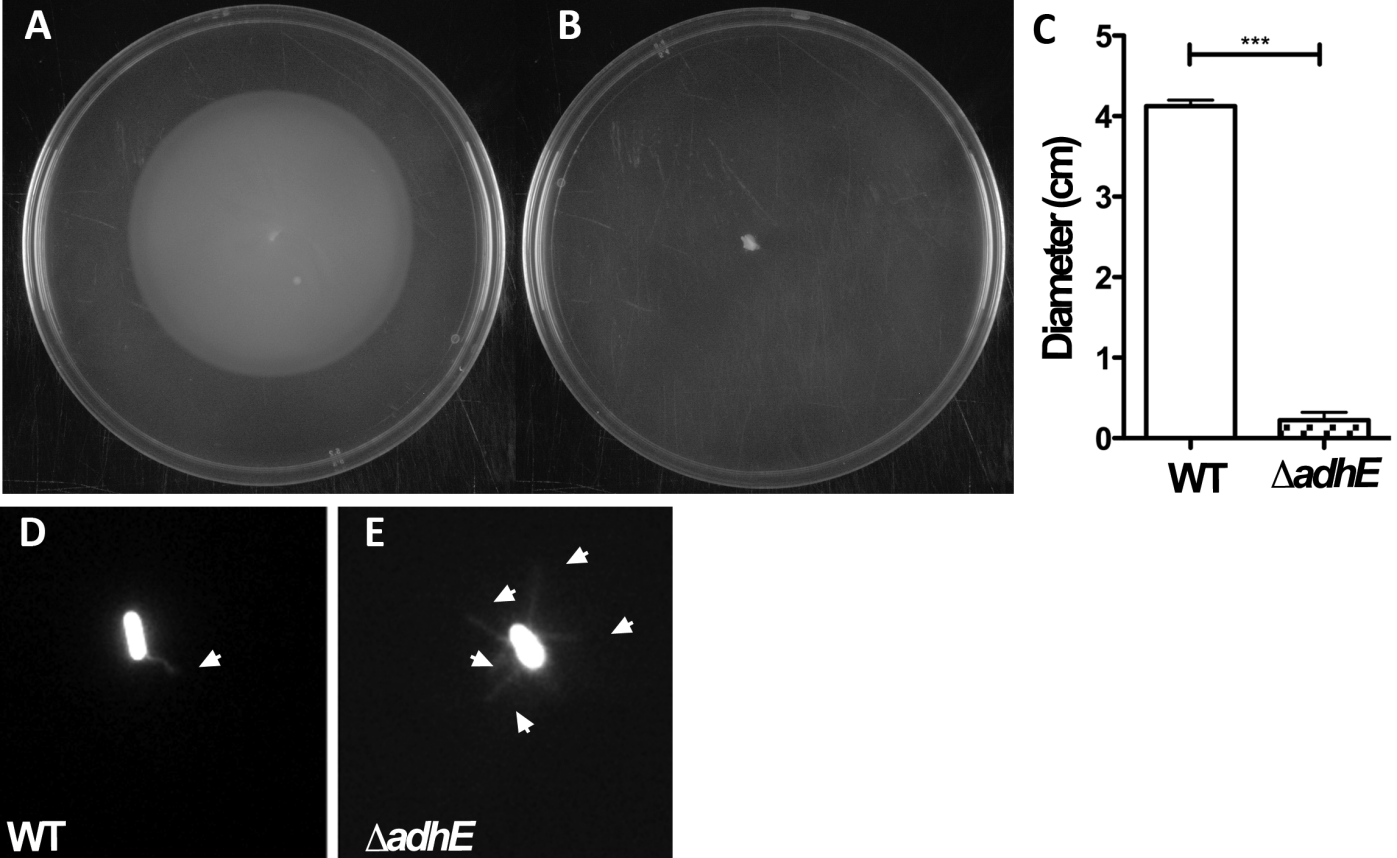
Deletion of *adhE* affects motility in *E. coli* O157:H7. Cultures of WT (A) and Δ*adhE* (B) strains were spotted onto 0.25% TB agar and incubated for 18 h at 30°C. Histogram to show the distance measured from the site of inoculation from three replicate experiments (C). The asterisks indicate the degree of significance as calculated using a Student's *t*-test and ‘***’ indicates a *P*-value below 0.001. Immunofluorescence microscopy images from real-time movies (Movie S1) examining bacterial motility in WT EHEC (D) and Δ*adhE* strains (E); flagella are indicated by white arrows. Scale bars are 2 μM.

Given that the Δ*adhE* mutant exhibited multiple apparently intact flagella, we wondered whether the mutant also displayed enhanced motility. Instead, the Δ*adhE* mutant was unable to migrate through semi-solid agar (Wolfe and Berg, 1989) (Fig. 2A). Changing the composition of the agar and incubation at different temperatures had no effect; the mutant did not migrate (data not shown). To determine whether this lack of migration resulted from a lack of motility, we visualized bacterial behaviour following culture in tryptone broth (TB) at 30°C, conditions that normally promote motility. Previous work has extensively characterized *E. coli* motility, which consists of directional ‘swimming’ that is interspersed by brief periods of ‘tumbling’ to facilitate chemotaxis (Porter *et al.*, 2011). We used AlexaFluor 488 dye esters to assess the arrangement of the flagellar filaments on the bacterial cell surface (Turner *et al.*, 2000). This enabled us to assess both swimming behaviour and flagellar motion. A polarized flagellar bundle, required for normal swimming activity, was visualized for WT EHEC (Fig. 2B and Movie S1). Transforming the WT with a plasmid encoding red fluorescent protein (RFP) enabled imaging for extended periods and showed both swimming and tumbling (Movie S2). In contrast, the Δ*adhE* mutant displayed a ‘paralysed’ phenotype (Fig. 2C and Movie S3). The flagella were dispersed over the surface of the bacterium, as expected of a peritrichous organism. Unlike WT flagella (Fig. 2B), the *adhE* flagella did not form a bundle (Fig. 2C). Collectively, these data show that the Δ*adhE* mutant can express and assemble flagella, but imply that the assembled flagella do not rotate.

### Deletion of *adhE* reduces binding to host cells and increases activation of TLR-5

**Fig 3:**
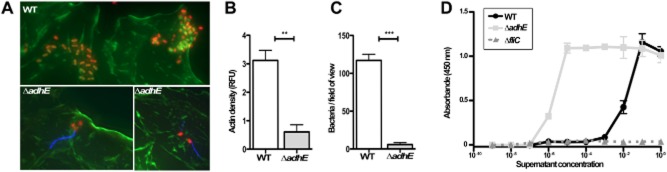
Deletion of *adhE* affects interactions with host cells. (A) Immunofluorescence images of A/E lesion formation for WT and Δ*adhE* bacteria following contact with Caco-2 cells. Bacterial strains were transformed with an RFP expressing plasmid (pRFP, red). Host cell actin was visualized using with FITC-phallodin (green). Flagella were detected using an anti-H7 primary and visualized with a secondary AlexaFluor 647 secondary conjugate (blue). Scales bars are 5 μM. (B) A/E formation was quantified using Volocity suite software (Perkin Elmer) by analysing the number of bacteria attached to the host cells and the density of actin beneath attached bacteria (C). Asterisks indicate the level of significance calculated from a Student's *t*-test: ‘**’ indicates a *P*-value between 0.001 and 0.01; ‘***’ indicates a P-value below 0.001. The hTLR5 response generated in response to WT and Δ*adhE* bacteria was tested using HEK-Blue hTLR5 cells. Secreted proteins from WT EHEC, Δ*adhE* and Δ*fliC* were added to HEK-Blue hTLR5 reporter cells and the level of NF-κB stimulation measured using Quanti-blue reagent, which reports the levels of SEAP through a change in absorbance at 450 nm. A representative graph from one of four replicate experiments is shown (D).

When added to epithelial cells, WT EHEC bacteria use their T3SS to condense host cell actin, facilitating close attachment – a process known as attaching and effacing (A/E) lesion formation. This process was visualized using immunofluorescence microscopy (Fig. 3A). After 4 h incubation of bacteria with host cells, WT bacteria clearly produced A/E lesions, a phenotype that was absent in the Δ*adhE* mutant. Indeed, both the total number of attached bacteria and the condensation of host cell actin were significantly reduced (*P* < 0.005) in the Δ*adhE* mutant relative to its WT parent (Fig. 3B and C). These results confirm that the mutant does not express the T3SS. In contrast to the WT, the Δ*adhE* mutant retained its flagella. Thus, it did not perform the normal switch from flagellar to T3SS expression, even in the presence of host cell signals or following direct contact with host cells.

Flagellin plays an important role in the activation of innate immune responses raising the possibility that the Δ*adhE* mutant might increase activation of TLR-5 signalling. This signalling was assessed using a HEK-293 TLR-5 cell line that produces secreted alkaline phosphatase in response to appropriate stimuli. Supernatants from WT EHEC, the Δ*adhE* mutant, and a Δ*fliC* mutant were added to the reporter cells across a range of dilutions and the extent of TLR-5 stimulation determined. The Δ*adhE* mutant exhibited a greater than 100-fold increase in TLR-5 stimulation compared with WT EHEC (Fig. 3D). The specificity of the reporter was confirmed by addition of supernatants prepared from a Δ*fliC* mutant; they produced no detectible TLR-5 activity (Fig. 3D).

### Deletion of *adhE* affects colonization and clinical disease *in vivo*

**Fig 4:**
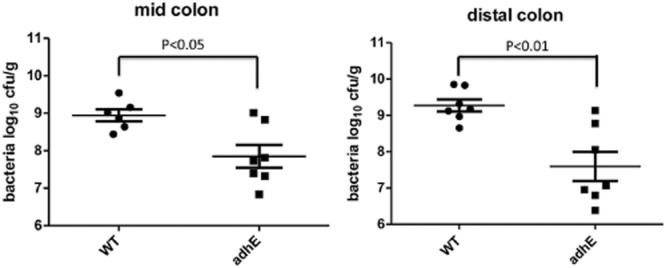
Deletion of *adhE* affects bacterial colonization *in vivo*. Recovery of TUV (•) and Δ*adhE* (▪) (cfu) from intestinal segments from infected rabbits. Numbers of bacterial cfu were determined in sections taken from the mid-colons (A), and distal colons (B) of rabbits 7 days post inoculation. The average and standard deviation are indicated for each experimental group.

Given that the *adhE* mutant dysregulated expression of both the T3SS and flagella, we tested its ability to colonize the mammalian intestine, using the infant rabbit model of *E. coli* O157:H7 infection (Ritchie *et al.*, 2003). In this model, *E. coli* O157:H7 colonization is dependent on a functional T3SS system (Ritchie *et al.*, 2003; Ritchie and Waldor, 2005). Furthermore, rabbits develop diarrhoea and intestinal inflammation when infected by WT EHEC, but not by mutants lacking intimin or Tir. Thus, EHEC pathogenesis also depends on the T3SS (Ritchie *et al.*, 2003). Litters of 3-day-old New Zealand White infant rabbits were oro-gastrically inoculated with either WT EHEC or the *adhE* mutant. Rabbits were scored for diarrhoea and the number of cells present in the intestine enumerated at 5 days post infection. As expected, all rabbits infected with WT EHEC developed severe diarrhoea, as evident by the presence of extensive areas of faecal staining on their hind legs and tails. In contrast, approximately 70% of the rabbits infected with the *adhE* mutant did not develop any visible signs of diarrhoea and the remaining animals exhibited only mild diarrhoea (*P* < 0.05) (Table 1). Furthermore, significantly fewer bacteria were recovered in intestinal homogenates obtained from rabbits infected with the *adhE* mutant compared with rabbits given WT EHEC. The number of organisms recovered from *adhE*-infected rabbits was > 10-fold less in the mid-colon (*P* < 0.05) and distal colon (*P* < 0.01) at 5 days post infection (Fig. 4A and B). Thus, the *adhE* mutant is less able to colonize the mammalian intestine and cause disease than WT EHEC. We conclude that AdhE plays a key role in virulence.

**Table 1:** Diarrheal status of rabbits infected with WT EHEC or the *adhE* mutant

Diarrhoea^a^	WT	Δ*adhE*
Severe	7	0
Mild	0	2
None	0	5
Fishers exact test	*P* < 0.05	

a Diarrhoea is scored as follows: severe – extensive contamination of ventral surfaces with liquid faeces; mild – localized areas of fecal contamination and observation of soft, formed faecal material; none – no fecal staining on animals' ventral surfaces with hard, formed stool occasionally present.

### Deletion of *adhE* increases transcription of the flagellar regulon

**Fig 5:**
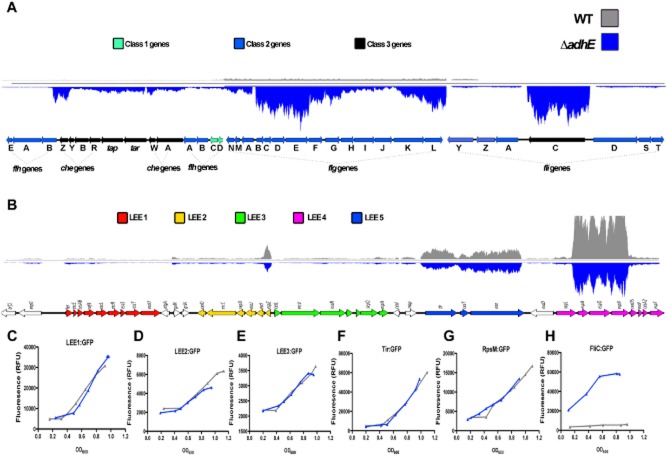
Transcriptional effects caused by deletion of *adhE* in *E. coli* O157:H7.A. RNA sequence analysis of the expression of the flagella operons of WT EHEC and Δ*adhE* bacteria cultured under T3SS-inducing conditions. Read density plots for WT and Δ*adhE* strains are shown to scale in grey and blue respectively. Flagella genes are coloured to indicate their class; 1: green; 2: blue; 3: black.B. RNA-seq analysis of the LEE, the five operons are colour coded for clarity as indicated by the key. To verify the RNA-seq analysis, transcription from key promoters was assessed using GFP reporter plasmids.C–F. Fluorescence levels from *LEE1* (C), *LEE2* (D), *LEE3* (E) and *LEE5* (F) were monitored in WT and Δ*adhE* strains during growth in T3SS-inducing conditions.G and H. As controls, fluorescence from GFP produced by the *rpsM* (G) and *fliC* (H) promoters were used.

To investigate the molecular basis for this phenotype, we used RNA-sequence analysis (RNA-seq), which provided us with a global view into the differences in gene expression between the Δ*adhE* mutant and its WT. The results were extremely specific. Members of the flagellar regulon were the most markedly affected genes (Fig. 5A). Transcripts from all three flagellar gene classes were significantly upregulated in the mutant (*P* < 0.005). This was true of the regulatory genes *flhC* (14-fold), *flhD* (16-fold) and *fliA* (5134-fold), of structural components such as *fliF* (138-fold), *flgE* (503-fold) and *fliC* (2143-fold), of the chemotaxis machinery, such as *cheA* (502-fold) and *cheY* (207-fold), and of the energy transduction system *motA* (384-fold) and *motB* (319-fold) (Table S1). In contrast, expression of several ‘housekeeping’ controls was similar in the Δ*adhE* mutant and its WT parent (Table S1). These results support the conclusion that the Δ*adhE* mutant synthesizes and assembles flagella.

### Deletion of *adhE* does not affect transcription of the LEE

Surprisingly, transcription of the *LEE* was not significantly (*P* < 0.05) affected in the Δ*adhE* background, with levels equivalent to those of the WT parent (Fig. 5B). Of the 42 *LEE*-encoded genes, 36 showed less than a twofold change in expression when the WT and *adhE* mutant were compared (Table S1) and none of these differences were statistically significant. These results were verified using reporter plasmids that carried four key *LEE* promoters (*LEE1*, *LEE2*, *LEE3* and *LEE5*) fused to *gfp*. As a control, we monitored expression of *rpsM*, which encodes a small ribosomal protein. When cultured under T3SS-inducing conditions, all four of the *LEE*-encoded promoters transcribed at similar levels in both the WT parent and its Δ*adhE* mutant, as did the ribosomal control (Fig. 5C–G). In contrast, transcription from the *fliC*::*gfp* reporter was strongly elevated in the Δ*adhE* mutant, consistent with the RNA-seq and immunoblotting data (Fig. 5H). Thus, the *adhE* mutant exhibits a fascinating phenotype: strong transcriptional upregulation that produces assembled but non-functional flagella coupled with post-transcriptional regulation of the *LEE*.

### Suppression of the T3SS is controlled post-transcriptionally in the *adhE* mutant

**Fig 6:**
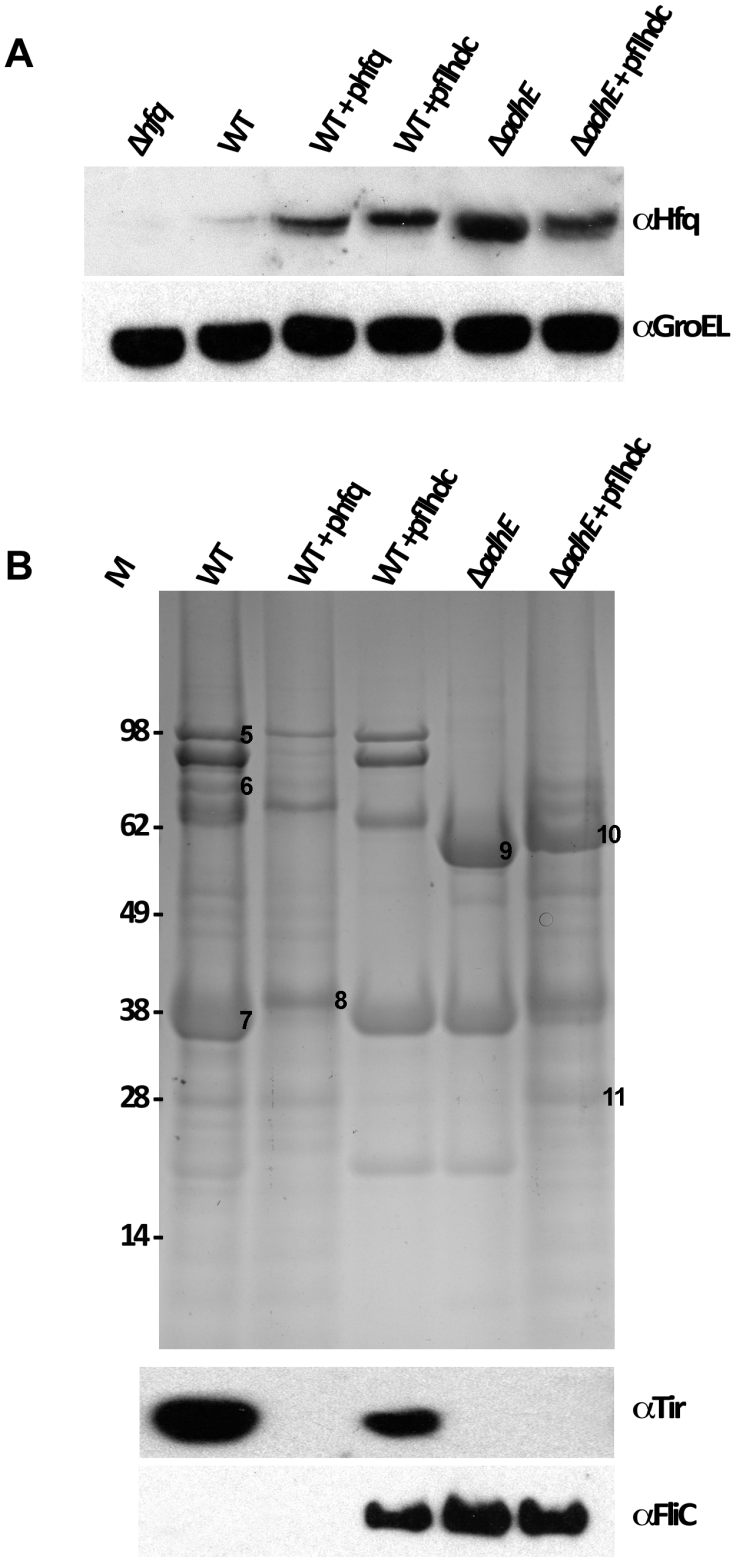
Post-translational regulation of the LEE is mediated via Hfq in the *E. coli* O157:H7 Δ*adhE* mutant.A. Immunoblotting using Hfq-specific antibodies showed elevated expression of Hfq in the Δ*adhE* strain compared with the WT. Expression of a plasmid expressing the flagellar specific regulators *flhC* and *flhD* also caused an increase in Hfq levels. The Δ*hfq* mutant acted as a control for antibody specificity and GroEL acts as a loading control.B. To examine the role of Hfq overexpression in regulation of the T3SS, proteins from the secreted fraction were prepared and visualized using Coomassie blue. Selected proteins (numbered) were removed and identified by tandem MS analysis as follows: 5, EspP; 6, intimin; 7 and 8, EspB; 9–11, flagellin. In the WT background, overexpression of either directly via a plasmid (phfq) or indirectly via flagella expression (pflhDC) results in a decrease of T3SS. The corresponding immunoblot shows the same samples probed for Tir and FliC.

In the Δ*adhE* mutant, there was a stark contrast between expression of the LEE, which was at WT levels, and T3SS protein secretion, which was strongly inhibited. Several studies have shown that the expression of the LEE can be controlled in a post-transcriptional manner. One key regulator that has been shown to affect *LEE* expression is Hfq, a chaperone that binds small regulatory RNA (sRNAs) and mRNAs to facilitate translational regulation in response to a variety of stresses. We therefore investigated Hfq levels by immunoblotting and found that Hfq expression was markedly increased in the *adhE* mutant (Fig. 6A), eightfold as determined by scanning densitometry of the blots. This result suggested that elevated levels of Hfq might be central to the regulation of the T3SS. To test this hypothesis, we raised Hfq levels in the WT parent by transformation with a plasmid that expresses Hfq, and found that secretion of T3SS effector proteins decreased (Fig. 6B). These included intimin (encoded by *LEE5*), EspB (encoded by *LEE4*) and EspP (encoded by pO157) (Fig. 6B). However, overexpression did not result in a complete loss of T3SS secretion and did not cause a switch to flagella expression, as seen in the *adhE* mutant. These data raised the possibility that Hfq inhibits production of the T3SS in the Δ*adhE* mutant in response to strongly elevated flagellar expression. To test this hypothesis, we transformed the WT strain with a plasmid carrying both *flhD* and *flhC*, thereby creating a scenario similar to the *adhE* mutant: concurrent transcription of both the flagella regulon and the LEE operons. Expression of *flhDC* in a WT background raised Hfq levels (Fig. 6A) and reduced production of the effector proteins from the T3SS compared with the WT, especially intimin and Tir (Fig. 6B). However, *flhDC* overexpression did not result in a complete switch to flagella expression observed in the *adhE* mutant.

### Deletion of *adhE* affects acetate production

**Fig 7:**
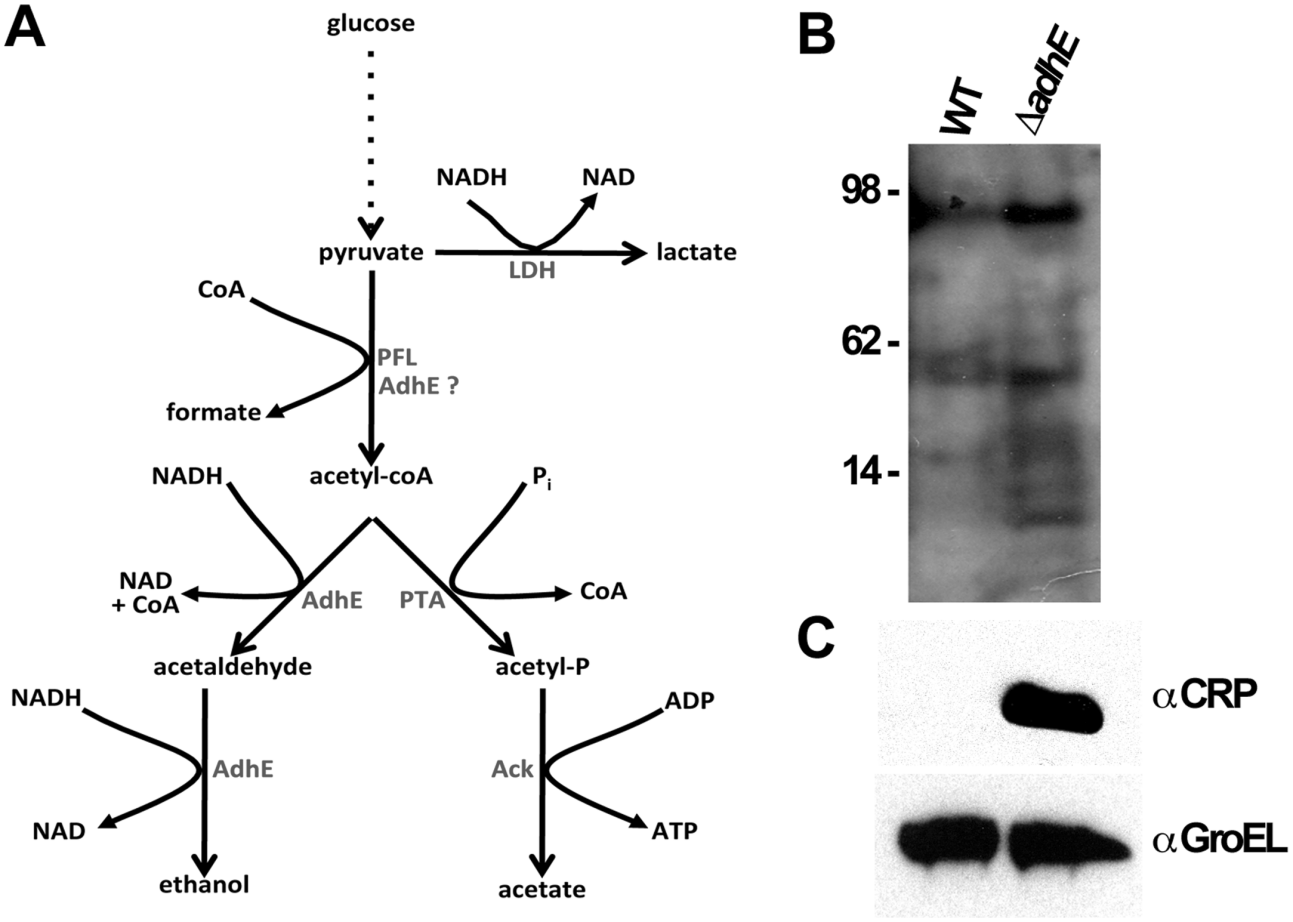
AdhE is central to acetate production and affects protein acetylation.A. AdhE encodes a bi-functional acetaldehyde-CoA dehydrogenase and alcohol dehydrogenase.B. Probing whole cell lysates with an anti-acetyl lysine antibody shows that deletion of *adhE* resulted in increased acetylation of several proteins.C. Immunoblotting for the cAMP receptor protein (CRP) in WT EHEC and the Δ*adhE* mutant reveals marked upregulation, an anti-GroEL antibody has been used as a loading control.

AdhE performs two enzymatic functions: the conversion of acetyl-CoA to acetaldehyde, followed by the conversion of acetaldehyde to ethanol (Fig. 7A). AdhE works in conjunction with the Pta-AckA pathway to recycle NAD^+^ and CoA with the generation of ATP by substrate phosphorylation (Wolfe, 2005). Deletion of AdhE would presumably affect levels of the metabolites directly associated with these two pathways, such as acetyl-CoA, acetyl phosphate and acetate. Thus, we hypothesized that virulence factor dysregulation might be related to changes in one or more of these metabolites. Using highly quantitative ^1^H-NMR, extracellular acetate levels were assessed in WT EHEC and its Δ*adhE* mutant. In the WT, extracellular acetate was measured at 4.41 ± 0.04 mM, a level that was raised by 18% in the mutant to 5.21 ± 0.05 mM. Because acetyl-CoA and acetyl phosphate are high energy central metabolites (Wolfe, 2005) that can function as acetyl donors for Nε-lysine acetylation in *E. coli* (Hu *et al.*, 2010; Thao and Escalante-Semerena, 2011; Weinert *et al.*, 2013; Kuhn *et al.*, 2014), we also examined the acetylation status of the Δ*adhE* mutant and its parent. An anti-acetyl lysine immmunoblot showed greater cross-reactivity for several different bands in the Δ*adhE* mutant, suggesting that numerous proteins were acetylated compared with the WT control (Fig. 7B). Furthermore, the Δ*adhE* mutant expressed 100-fold more cAMP receptor protein (CRP) than its WT parent, as determined by scanning densitometry of triplicate immunoblots (Fig. 7C). This result is intriguing because acetate accumulation induces *flhDC* and thus flagellar expression via CRP (Soutourina *et al.*, 1999).

Finally, to test whether the metabolic effects of *adhE* deletion were specific to the AdhE and Pta-AckA pathways, we conducted a global metabolomic analysis using a mass-spectrometry based approach, which enabled the detection and relative quantification of more than 300 metabolites that originated from diverse cellular pathways, including amino acid synthesis, lipid and carbohydrate metabolism, and nucleic acid synthesis. No significant differences were detected in the Δ*adhE* mutant relative to its WT parent (data not shown), suggesting that deletion of AdhE specifically affects metabolites immediately associated with these two pathways.

## Discussion

That central metabolism plays a role in regulating virulence factors is becoming increasingly evident. From a biological viewpoint this makes sense, as the metabolic status of a cell should determine its ability to effectively adapt to environmental changes encountered during the course of an infection. An early report suggested that acetyl-CoA or a derivative was responsible for the control the T3SS of *Pseudomonas aeruginosa* (Rietsch *et al.*, 2005; Rietsch and Mekalanos, 2006), while a more recent report linked *Vibrio cholerae* virulence to acetyl-CoA (Minato *et al.*, 2013). Several other reports provided evidence that other metabolic products induce expression of virulence factors, including the Shiga toxin and flagella (Fukuda *et al*., 2011; 2012,; Tobe *et al.*, 2011). Deletion of AdhE, a metabolic enzyme linked to acetyl-CoA, offered an opportunity to further these findings.

The anaerobic role of AdhE is well-characterized; it is essential for ethanol fermentation (Clark and Cronan, 1980). Since AdhE also is expressed under aerobic conditions (Echave *et al.*, 2003), its role is likely not limited to anaerobic conditions, but the nature of that aerobic role has remained unknown. Here, we deleted *adhE* and observed a novel pleiotropic phenotype: (i) overexpression of the flagellar regulon that results in assembled but non-functional flagella coupled to (2) a complete lack of the T3SS, including the structural proteins that comprise the secretory apparatus and its effector proteins. Thus, the mutant was immotile. It also stimulated activation of innate immune responses through TLR-5, presumably because of the flagella that it assembles on its surface. Finally, it bound to host cells in fewer numbers, likely because it lacks the T3SS. Collectively, these phenotypes rendered the Δ*adhE* mutant a less effective pathogen. In fact, when tested *in vivo* using the rabbit model of EHEC infection, the Δ*adhE* mutant colonized poorly and caused less diarrhoea than the WT, traits that are dependent on a functional T3SS (Ritchie *et al.*, 2003).

Deletion of *adhE* should perturb the pathways associated with acetyl-CoA. Indeed, ^1^H-NMR analysis showed that the Δ*adhE* mutant excretes about 20% more acetate than its WT parent. Since the undissociated form of acetate easily permeates the membrane and distributes according to the ΔpH across the membrane (Booth, 1985), this 18% difference corresponds to a 9 mM increase in the intracellular of acetate pool, from 35 mM in the WT to 46 mM in the Δ*adhE* mutant. These calculations assume a ΔpH of 0.88 based on the media being buffered at pH 7 and an intracellular pH of 7.88 for *E. coli* (Booth, 1985). This difference could be physiologically significant. Indeed, as little as 5 mM acetate alters EHEC Shiga toxin translocation from the gut lumen to the bloodstream (Fukuda *et al*., 2011; 2012) and acetate accumulation is reported to induce *flhDC* transcription using a CRP-dependent mechanism (Soutourina *et al.*, 1999). Acetate also directly activates *fliA* and other Class II genes (Tobe *et al.*, 2011). These reports are relevant, as both CRP and σ^28^ (encoded by *fliA*) were elevated in the Δ*adhE* mutant.

The accumulation of acetate in the extracellular milieu suggests that deletion of *adhE* increases carbon flux through the Pta-AckA pathway. This increased flux should affect the intracellular pools of both acetyl-CoA and acetyl phosphate, high-energy molecules that can function as acetyl donors for protein acetylation (Hu *et al*., 2010; 2013; Weinert *et al.*, 2013; Kuhn *et al.*, 2014). Indeed, the increased signal detected using the anti-acetyl lysine antibody suggested that numerous proteins were strongly acetylated in the Δ*adhE* mutant compared with the WT control. While we have not yet determined the identity of the acetylated proteins, it should be noted that recent work has shown that acetylation is quite extensive in both *E. coli* (Zhang *et al.*, 2009; Zhao *et al.*, 2010; Wang, 2013; Weinert *et al.*, 2013; Kuhn *et al.*, 2014) and in *Salmonella enterica* (Q. Wang, 2013). The lack of motility by the *adhE* mutant might result from acetylation of one or more flagellar proteins. Alternatively, the flagellum might be assembled incorrectly, thereby blocking its function. A candidate for this role could be YcgR. This flagellar regulon member acts as a brake when bound by the second messenger cyclic-di-GMP (Boehm *et al.*, 2010; Paul *et al.*, 2010).

Our preferred model is that deletion of the AdhE pathway leads to increased flux through the Pta-AckA pathway, which leads to increased intracellular acetate, acetyl phosphate, and acetyl-CoA. The elevated intracellular acetate increases expression of CRP and σ^28^. CRP activates *flhDC* (which encodes the master regulator of the flagellar regulon), while σ^28^ boosts expression of Class III proteins, such as flagellin. The assembled flagella do not rotate either because a key component is misassembled, YcgR is overexpressed, and/or some flagellar component is inappropriately acetylated.

The other aspect of the phenotype was a lack of production of the T3SS, including the structural proteins required for secretion and the effector proteins themselves. Whereas flagella expression was activated at the level of transcription, it was clear that a different mechanism was inhibiting production of the T3SS. One important caveat of our study was that it was largely performed in media that induces expression of the T3SS. Therefore, the Δ*adhE* mutant, despite activating flagella expression, was still subject to the environmental signals that stimulated LEE transcription. This was shown very clearly by both RNA-seq and reporter fusion analyses. However, this led to a paradox: LEE transcription in the mutant was equivalent to that in the WT parent, yet expression or secretion of T3SS effector proteins was virtually undetectable.

One strong candidate protein with the capacity to sequester and turn over RNA species is Hfq. Binding of Hfq to an mRNA transcript can either promote its stabilization or degradation, depending on the specific interaction (Massé and Gottesman, 2002; Vytvytska *et al.*, 1998; Q. Wang, 2013). Deletion *of hfq* in *E. coli* O157 strain EDL933 leads to increased levels of LEE-encoded proteins by negatively controlling levels of the regulators GrlA and GrlR post-transcriptionally (Hansen and Kaper, 2009). It should be noted that Hfq-mediated effects can be quite strain specific and can be pleiotropic: deletion of *hfq* in *E. coli* O157 strain 86-24 led to quite different phenotypes, including a transcriptional downregulation of the LEE (Kendall *et al.*, 2011). The strains in this work were derivatives of EDL933 leading to the hypothesis that, in the Δ*adhE* mutant, an increase in Hfq expression could suppress LEE expression. While previous studies have focused on the role of Hfq on the master regulator Ler and GrlA (Hansen and Kaper, 2009), ongoing studies have revealed a much wider set of LEE encoded transcripts that can be affected by Hfq, including LEE4 and LEE5 (J. Tree and D. Gally, pers. comm.). This is consistent with our previous research showing that these particular operons are controlled post-transciptionally (Roe *et al*., 2003; 2004) and with data in the current work showing that Hfq regulation of T3SS in the Δ*adhE* mutant is a critical factor after Ler induction of the system. This is apparent from the marked phenotypic impact of the Δ*adhE* mutant on the T3SS despite very little effect on transcript levels, including those induced by Ler (LEE2/3 and LEE5, Fig. 5). Immunoblotting confirmed that Hfq was elevated in the Δ*adhE* mutant. Mimicking the concurrent transcription of flagella and LEE operons by overexpression of *flhDC* in a WT background also raised Hfq levels. These data suggest that Hfq acts as a final ‘safety net’ to mop up transcripts that are expressed, but selectively not translated into proteins.

Our finding that dysregulation of flux from the acetyl-CoA pool regulates the switch from the motile flagellated state to the attached T3SS-expressing state may be help explain why EHEC display a tropism to one distinct site in the bovine host. Previous work has shown that EHEC use their T3SS to colonize the recto-anal junction of cattle (Naylor *et al.*, 2003). Cattle contain very high concentrations of short chain fatty acids (SCFAs, e.g. acetate) that vary along the gastrointestinal tract. In the bovine rumen, it is common for acetate concentrations to vary from 60 to 150 mM, with levels at their lowest in the stomach and at the rectum (Bergman, 1990). It is attractive to propose that the low levels of acetate present at the rectum correspond with expression of the T3SS and colonization of the recto-anal tissue. Overall, it seems clear that the relevance of both host and bacterial-derived metabolites can play a major role in influencing virulence gene expression and that this markedly affects the interaction of the pathogen with its host. Recent work has also shown that the effects of Shiga toxin can be modulated *in vivo* by manipulation of acetate and butyrate levels (Fukuda *et al.*, 2012; Zumbrun *et al.*, 2013). It is worth noting that the strains used in this study were Shiga toxin negative; therefore, toxin activity would not have influenced the clinical symptoms observed in our experiments.

In summary, we have shown that expression of AdhE, a bi-functional acetaldehyde-CoA dehydrogenase and alcohol dehydrogenase, is central for appropriate expression of the genes for motility and attachment in *E. coli* O157:H7 both *in vitro* and *in vivo*. The molecular basis to the phenotype was found to be multifactorial involving perturbations to acetate levels that directly influenced flagella expression, post-transcriptional regulation of the T3SS through Hfq, and effects on protein acetylation. The importance of AdhE for appropriate expression of virulence genes paves the way for further studies to specifically target this protein.

## Experimental procedures

### Strains and media

The *E. coli* O157:H7 wild-type strain used in this study was TUV93-0 (Campellone *et al.*, 2004). The Δ*adhE* mutant was generated using allelic exchange (Emmerson *et al.*, 2006). To verify that all phenotypes were due to the mutation, it was subsequently repaired by allelic exchange of the WT *adhE* allele back into the chromosome at the native locus (Δ*adhE* + *adhE*). Bacteria were cultured overnight in LB media and the T3SS induced by culturing in MEM-HEPES (Sigma). Motility assays were carried out using tryptone broth (1% tryptone and 0.5% sodium chloride) with 0.25% agar. Bacteria were inoculated onto the centre of the plate and incubated at 34°C overnight (Wolfe and Berg, 1989).

### Animal protocols

Experiments were performed as described previously (Ritchie and Waldor, 2005) using the TUV93-0 strain and the Δ*adhE* mutant. Bacterial doses of 5 × 10^8^ colony-forming units (cfu) per 90 g of rabbit body weight were used in the infections. Post inoculation, the infant rabbits were weighed daily and observed twice daily for clinical signs of illness. Diarrhoea was scored as follows: ‘none’, no fecal staining on animals' ventral surfaces with hard, formed stool occasionally present; ‘mild’, localized areas of faecal contamination and observation of soft, formed faecal material; ‘severe’ extensive contamination of ventral surfaces with liquid faeces. To limit any litter-specific effects, two different litters were used to test each type of inoculum studied.

### Ethics statement

This study was carried out in strict accordance with the recommendations in the United Kingdoms Home Office Animals (Scientific Procedures) Act of 1986, which outlines the regulation of the use of laboratory animals for the use of animals in scientific procedures. The experiments described were subject to approval by the University of Surrey Ethics Committee and by a designated Home Office Inspector. All experiments were subject to the 3 R consideration (refine, reduce and replace) and all efforts were made to minimize suffering.

### RNA sequencing

Total RNA was prepared as described previously (Tree *et al.*, 2009) and depleted for ribosomal sequences using a Microbexpress kit (Ambion). Sequencing of cDNA was carried out on an Illumina Genome Analyser IIx using single ended reads and 6 samples per lane. Raw transcript data were analysed using CLC Genomics Workbench 4 using the E. coli EDL933 genome as a reference. The sequence reads reported in this paper have been deposited in the European Nucleotide Archive under study PRJEB6365 (ERS462727-ERS462730). Data were normalized using DESeq in R/Bioconductor (Anders and Huber, 2010). Coverage graphs were generated using EasyFig 2.1 (Sullivan *et al.*, 2011). Experiments consisted of 3 biological replicates of EHEC WT samples to allow variance in gene expression to be calculated. Comparison with the Δ*adhE* mutant data allowed significantly differentially expressed genes to be identified.

### Reporter fusions

Expression from reporter plasmids was performed as described previously (Roe *et al.*, 2004) using plasmids pAJR70 (promoterless control), pAJR71-75 (*LEE1*-*5* fused to *gfp*), pAJR199 (*fliC*::*gfp*) and pAJR145 (*rpsM*::*gfp*). Transformants were grown overnight in LB media with appropriate antibiotics then the next morning diluted to an OD_600_ of 0.08 in MEM-HEPES (Sigma). Cultures were shaken at 200 rpm in Erlenmeyer flasks incubated at 37°C. At intervals, 1ml of culture was removed from the flask and 200 μl aliquots were analysed in triplicate with a fluorescent plate reader (Fluorstar Optima; BMG) at 37°C. Fluorescence was plotted against OD_600_ using Microsoft Excel software.

### Protein characterization

Secreted proteins were prepared as described in Roe *et al*. (2003), briefly, bacteria were cultured in MEM-HEPES to OD_600_ = 0.8 and cells were pelleted by centrifugation (10 min at 3000 *g*). The secreted proteins in the supernatant were precipitated by addition of 10% TCA, pelleted by centrifugation (40 min at 10 000 g) and resuspended in 1.5 mM Tris pH 8.8. Bacterial cell pellets were lysed using Bugbuster (Millipore). Whole cell and secreted proteins separated using SDS-PAGE and stained with Coomassie brilliant blue or transferred to nitrocellulose (Amersham) for immunoblotting. Antibodies for the T3SS proteins and Hfq were provided by Prof David Gally (University of Edinburgh) and Profs Susan Gottesman (Centre for Cancer Research, Bethesda) and Elisabeth Sonnleitner (University of Vienna) respectively. The FliC (Mast Assure), GroEL (Enzo), Sigma factors (Neoclone) and anti acetyl lysine antibodies (Abcam) were purchased and used as indicated by the manufacturer. Bands from the gel were excised to permit in-gel trypsin digestion and analysis by liquid chromatography electrospray ionization tandem mass spectrometry (Shevchenko *et al.*, 2006).

### Microscopy

Real-time flagellar imaging was performed as described (Turner *et al.*, 2000) on a Zeiss M1 Axioimager microscope using Volocity suite software (Perkin Elmer). T3SS, flagella expression and formation of A/E lesion were analysed as described previously (Tree *et al.*, 2009).

### Measurement of acetate concentrations

One-dimensional high-resolution ^1^H-NMR spectra were acquired on a Brucker AVANCE 600 MHz spectrophotometer at 298 K. The acetate peak at ∼ 1.9 ppm was identified and integrated between 1.921 and 1.888 ppm for each sample. This value was compared with a standard curve of known acetate concentrations to quantify the levels in the media. Measurements from triplicate independent experiments were averaged and differences analysed by an unpaired *t*-test.

### HILIC-orbitrap analysis metabolites

Bacterial cultures were grown to mid-log phase and 1 × 10^6^ cells and rapidly cooled to 4°C to quench metabolism by submersion of the flask in a dry ice-ethanol bath. The cold cell culture was centrifuged at 1250 RCF for 10 min and the supernatant completely removed. Cell lysis and protein denaturation was achieved by addition of 200 μl of 4°C chloroform: methanol: water (ratio 1:3:1) followed by vigorous shaking for 60 min at 4°C. Extract mixtures were centrifuged for 2 min at 16 000 RCF, 4°C. The supernatant was collected, frozen and stored at −80°C until further analysis.

Samples were analysed using an UltiMate 3000 RSLC (Thermo Fisher) with a 150 × 4.6 mm ZIC-pHILIC column running at 300 μl min^−1^ coupled to an Orbitrap Exactive (Thermo Fisher) mass spectrometer. The gradient ran from 20% H_2_O 80% acetonitrile to 80% H_2_O, 80% acetonitrile in 15 min, followed by a wash at 5% acetonitrile, 95% H_2_O for 4 min, and equilibration at 20% H_2_O, 80% acetonitrile for 6 min. Raw mass spectrometry data was processed using an in-house pipeline, consisting of XCMS (Smith *et al.*, 2006) (for peak picking), MzMatch (Scheltema *et al.*, 2011) (for filtering and grouping) and IDEOM (for further filtering, post-processing, statistical analysis and identification). Core metabolite identifications were validated against a panel of unambiguous standards by mass and retention time. Additional putative identifications were assigned by mass and predicted retention time (Creek *et al.*, 2011).
