# Supplementary material for: The metabolic enzyme AdhE controls the virulence of Escherichia coli O157:H7
Source: Mol Microbiol. 2014 Jun 9;93(1):199–211. doi: 10.1111/mmi.12651 (PMC4249723; doi:10.1111/mmi.12651)
Supplement: Supplementary file 1 — Supporting Information [file mmi0093-0199-SD1.pdf]

Figure S1

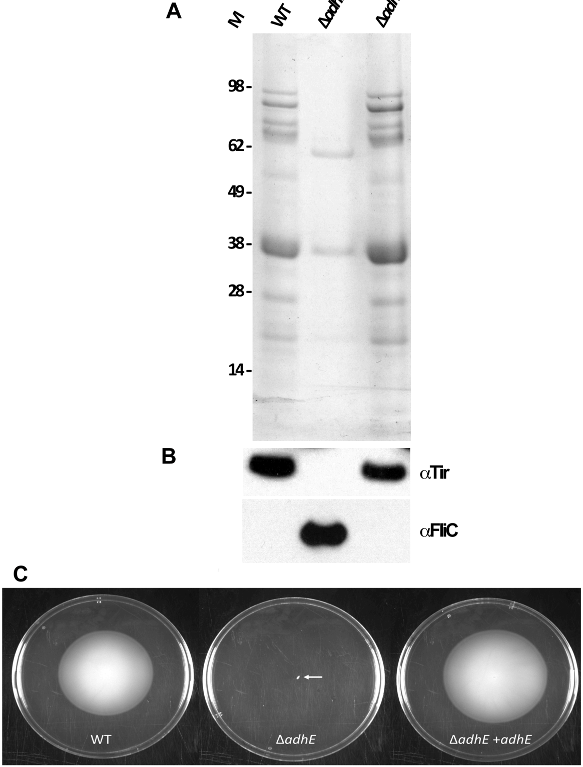
A
M
WT
ΔadhE
ΔadhE+adhE
98
62
49
38
28
14
B
αTir
αFliC
C
WT
ΔadhE
ΔadhE +adhE

Table S1: Motility associated genes significantly affected in the Δ*adhE* mutant compared with the WT control. As a comparison, the expression levels of several “housekeeping” genes are also provided.

	Normalized Read counts		
**Gene**	**WT**	**ΔadhE**	**Fold Increase (p< 0.005)**
Class 1			
flhC	11.885	167.623	14.1
flhD	10.676	168.421	15.8
Class 2			
flgN	1.416	71.838	50.7
flgM	1.291	79.022	61.2
flgA	4.016	201.946	50.3
flgC	0.127	405.488	3192.8
flgD	0.646	859.666	1330.8
flgE	3.616	1819.107	503.1
flgF	1.031	597.057	579.1
flgG	1.295	498.079	384.6
flgH	0.910	185.183	203.5
flgI	0.646	189.174	292.8
flgJ	1.290	90.995	70.5
flgK	6.206	903.567	145.6
flgL	11.249	990.571	88.1
flhA	8.153	121.327	14.9
flhB	2.191	66.251	30.2
fliY	219.270	750.312	3.4
fliZ	0.779	311.300	399.6
fliA	0.127	652.133	5134.9
fliD	1.792	1066.401	595.1
fliS	1.158	324.071	279.9
fliT	2.078	206.735	99.5
fliF	1.303	179.596	137.8
fliG	1.285	179.596	139.8
fliH	1.556	112.547	72.3
fliI	1.285	81.417	63.4
fliK	3.241	57.471	17.7
fliL	1.040	55.874	53.7
fliM	1.024	135.695	132.5
fliN	0.516	60.664	117.6
fliO	0.127	11.175	88.0
fliP	1.301	20.753	16.0
Class 3			
fliC	18.257	39130.360	2143.3
tar	4.921	1886.156	383.3
cheW	3.617	742.330	205.2

cheA	3.208	1609.978	501.9
motB	0.639	203.542	318.5
motA	0.761	292.941	384.9
cheZ	6.477	1650.686	254.9
cheY	4.792	994.562	207.5
cheB	3.235	597.855	184.8
cheR	1.546	556.348	359.9
ycgR	2.312	225.094	97.7
**Housekeeping**			Fold-change >0.005
groEL	225.738	179.12	1.2
gapA	793.805	1,106.77	0.7
dnaJ	18.085	19.77	0.9
gyrA	30.275	34.54	0.8
arcA	91.750	97.03	0.9
rrsA	7550.233	6,636.51	1.1
fumC	5.588	3.89	1.4
rpoS	666.005	451.89	1.4
recA	76.333	82.51	0.9
**T3SS**			
espF	123.57	90.12	1.37
Z5102	139.34	109.35	1.27
escF	177.31	115.02	1.54
Z5104	886.29	494.59	1.79
espB	4564.53	1851.13	2.47
espD	3560.95	1545.96	2.30
espA	4267.20	1967.13	2.17
sepL	139.43	83.53	1.67
escD	33.61	20.64	1.63
eae	775.99	664.49	1.17
Z5111	457.86	478.78	0.96
tir	722.28	407.76	1.77
Z5113	160.87	80.47	2.00
Z5114	44.10	30.66	1.44
Z5115	110.16	50.69	2.17
sepQ	41.51	30.71	1.35
Z5117	12.44	15.62	0.80
Z5118	20.97	14.91	1.41
escN	45.31	28.32	1.60
escV	30.54	13.33	2.29
Z5121	5.72	1.87	3.06
sepZ	871.51	445.48	1.96
Z5123	99.65	55.27	1.80
escJ	36.81	14.18	2.60
Z5125	34.64	39.27	0.88
escC	42.39	33.62	1.26
cesD	43.05	33.82	1.27
Z5128	46.87	27.23	1.72
Z5129	84.39	66.41	1.27
Z5131	5.12	5.06	1.01

escU	4.25	4.15	1.02
escT	4.66	2.99	1.56
escS	7.21	4.30	1.68
escR	9.60	8.87	1.08
Z5136	30.68	16.45	1.87
Z5137	51.61	39.24	1.32
Z5138	47.71	35.82	1.33
Z5139	44.93	32.56	1.38
Z5140	63.58	72.28	0.88
Z5142	47.61	27.15	1.75
Z5143	3.27	2.02	1.62
Housekeeping			
groEL	225.73	179.12	1.2
gapA	793.80	1,106.77	0.7
dnaJ	18.08	19.77	0.9
gyrA	30.27	34.54	0.8
arcA	91.75	97.03	0.9
rrsA	7550.23	6,636.51	1.1
fumC	5.58	3.89	1.4
rpoS	666.00	451.89	1.4
recA	76.33	82.51	0.9
